# Insights Into Manganese Solubilizing *Bacillus* spp. for Improving Plant Growth and Manganese Uptake in Maize

**DOI:** 10.3389/fpls.2021.719504

**Published:** 2021-11-02

**Authors:** Ayesha Ijaz, Muhammad Zahid Mumtaz, Xiukang Wang, Maqshoof Ahmad, Muhammad Saqib, Hira Maqbool, Ahmad Zaheer, Wenqiang Wang, Adnan Mustafa

**Affiliations:** ^1^Institute of Molecular Biology and Biotechnology, The University of Lahore, Lahore, Pakistan; ^2^College of Life Sciences, Yan’an University, Yan’an, China; ^3^Department of Soil Science, The Islamia University of Bahawalpur, Bahawalpur, Pakistan; ^4^Institute of Soil and Environmental Sciences, University of Agriculture, Faisalabad, Pakistan; ^5^SoWa Research Infrastructure, Biology Centre CAS, České Budějovice, Czechia

**Keywords:** *Bacillus* spp., manganese oxide, manganese reducing bacteria, minerals solubilization, rhizobacteria, *Zea mays*

## Abstract

Manganese (Mn) is an essential micronutrient for plant growth that is involved in the structure of photosynthetic proteins and enzymes. Mn deficiency is widespread mainly in dry, calcareous, and sandy soil, which leads to a significant decrease in crop yield. Mn-reducing bacteria promote the solubilization of Mn minerals, thus increasing Mn availability in soil. The present study aimed to assess the Mn solubilizing ability and plant growth-promoting potential of *Bacillus* spp. strains for maize plants with insoluble Mn compounds. Several Mn-solubilizing bacterial (MSB) strains were isolated from the maize rhizosphere using nutrient agar media amended with 50 mM MnO_2_. These strains were screened based on qualitative and quantitative solubilization of Mn, phosphorus, potassium, and zinc and production of ammonia. The majority of MSB strains were positive for catalase, protease, amylase, and oxidase activity, while more than 60% of tested strains were positive for lipase activity, and the production of indole-3-acetic acid and siderophores. Forty-five percent of the tested strains also showed solubilization of potassium. All the MSB strains were evaluated for their ability to promote plant growth and Mn uptake in the presence of MnO_2_ under axenic sand culture conditions. The results revealed that inoculation with MSB strains under sand culture significantly improved the growth of maize seedlings except for strains ASH7, ASH10, and ASH12. Comparatively, strains ASH6, ASH11, ASH19, ASH20, and ASH22 demonstrated a better increase in plant growth, fresh and dry biomass, and Mn uptake in roots and shoots than the other strains tested. All of these strains were identified as *Bacillus* spp. through 16S rRNA partial gene sequencing. Maize inoculation with these selected identified MSB strains also resulted in an increase in maize growth and nutrient uptake in maize roots and shoots under soil culture conditions in the presence of native soil Mn. The current study highlights the importance of MSB strain inoculation which could be a potential bioinoculants to promote plant growth under Mn deficiency.

## Introduction

Plant health is of ultimate significance to agricultural productivity and prominently depends on a series of processes affecting physiological functioning. Plants cope with changes in environmental and edaphic factors and nutrient deficiency by adjusting metabolite compositions (Schöttler and Tóth, 2014). According to the classical Liebig law of the minimum, deficiency of any essential nutrients cannot be compensated by any other nutrients, which is why a balanced supply of essential plant nutrients is required for optimal plant growth (Whitcomb et al., 2014).

Manganese (Mn) is an essential micronutrient with many functional roles in plant physiology, especially in electron transport in photosystem II (PSII), chloroplast structure, and N metabolism (Campbell and Nable, 1988). Mn acts as an activator and cofactor of various metalloenzymes and catalyzes various enzyme reactions including redox reactions, phosphorylation, decarboxylation, and hydrolysis (Schmidt and Husted, 2019). Mn activates more than 35 enzymes, including phenylalanine ammonia-lyase decarboxylases, dehydrogenases, and different glycosyltransferases (White et al., 1993). Mn deficiency causes oxidative stress and a reduction in photosynthetic electron transport, water use efficiency, and root functioning (Schmidt et al., 2016). Its deficiency causes a reduction in the wealth of PSII light-harvesting complex II supercomplexes and D1 together with PSII membrane extrinsic proteins PsbP and PsbQ (Schmidt et al., 2015). In aging mesophyll cells of wheat, Mn deficiency causes an increase in Mn-superoxide dismutase activity from base to tip in leaves due to the production of reactive oxygen species in peroxisomes or the respiratory chain in mitochondria (Leonowicz et al., 2018). In maize (*Zea mays* L.), a deficiency of Mn disturbs N metabolism by restricting NO_3_^–^ uptake and transportation by the production of inhibiting enzymes, *viz*. glutamine synthase, nitrate reductase, and glutamic-oxaloacetic transaminase cause a decline in chlorophyll synthesis and protein solubility (Gong et al., 2011).

Manganese biogeochemistry is complex in soil and exists in three oxidation states *viz*. Mn(II), Mn(III), and Mn(IV). Plants take up only the divalent form (Mn^2+^) of Mn, while Mn(IV) is highly insoluble and precipitates in soil solution. Mn availability to plants is greatly influenced by soil pH and redox conditions as an increase in soil pH causes a reduction in its availability by forming MnO_2_ complexes, while its availability increases under reduced O_2_ conditions in terms of soil compaction and flooding (Schmidt et al., 2016; Schmidt and Husted, 2019). Its deficiency symptoms frequently appear patchy due to the irregular distribution of Mn-oxidizing and reducing soil conditions. Globally, Mn deficiency is a widespread problem mainly in dry, calcareous, and sandy soils (Hebbern et al., 2005; Schmidt et al., 2013). A significant reduction in crop yield was observed in Mn-deficient regions, while Mn deficiency in severe winters caused complete crop loss (Schmidt et al., 2013). The agronomic effectiveness of various inorganic and organic Mn fertilizers is greatly influenced by their solubility in water, application method, soil properties, and nature of any macronutrient carrier. Manganous sulfate is mostly applied to the soil and in foliar sprays to address Mn deficiency in crops; however, its efficiency is lower in alkaline and dry soils (Martens and Westermann, 1991).

Manganese (IV) is reduced to Mn(II) through biological or chemical processes due to the presence of protons and electron-carrying reducing agents produced by plant roots, microorganisms, or through organic matter decomposition (Uren, 1981). Various Mn-reducing microorganisms, e.g., *Arthrobacter*, *Acinetobacter*, *Achromobacter*, *Aspergillus*, *Bacillus*, *Clostridium*, *Enterobacter*, *Lysinibacillus*, *Micrococcus*, *Pseudomonas*, and *Staphylococcus*, have been reported in the literature (Baglin et al., 1992; Das et al., 2012; Ghosh et al., 2016). Inoculation with Mn-reducing microorganisms, especially bacteria, can improve Mn uptake and plant growth in Mn-deficient soil (Huber and McCay-Buis, 1993). Mn-reducing bacteria can be termed Mn-solubilizing bacterial (MSB) that promote Mn dissolution through protonation of metal anions and production of organic acids that form a soluble complex of Mn ligands (Wei et al., 2012). Recently, Tang et al. (2020) reported plant growth promotion and Mn accumulation in *Myriophyllum verticillatum* through inoculation with Mn-resistant *Bacillus cereus* WSE01. Numerous other researchers also reported an increase in Mn availability, uptake and plant growth promotion through inoculation of various mycorrhizae and soil bacterial strains (Bromfield and David, 1976; Nogueira et al., 2007).

Therefore, we investigated the effect of various *Bacillus* strains possessing multiple plant growth-promoting (PGP) characteristics with importance for maize growth promotion and Mn uptake under Mn-insoluble compounds. We proposed that bacterial strains with the ability to solubilize Mn compounds *in vitro* and possess multiple PGP attributes could improve maize growth and Mn uptake compared to the uninoculated control in the presence of insoluble Mn compounds. Our main goals were to (i) evaluate the *in vitro* Mn-solubilizing ability of various isolated bacterial strains; (ii) determine the bacterial ability to solubilize phosphorus (P), potassium (K), and zinc (Zn) *in vitro*; (iii) screen the bacterial strains for *in vitro* PGP characterization; and (iv) assess the impact of Mn-solubilizing strain inoculation on growth and Mn uptake in maize plants in the presence of insoluble Mn compounds.

## Materials and Methods

### Isolation of Manganese-Tolerant Rhizobacteria

Manganese-tolerant rhizobacteria were isolated from the rhizosphere of maize grown in different farm fields located in Sargodha and Sheikhupura, Punjab, Pakistan. Rhizospheric samples were collected by uprooting maize seedlings in a sterile plastic polythene bag and transferred to the Laboratory of Microbiology, Institute of Molecular Biology and Biotechnology (IMBB), The University of Lahore (UOL), Lahore, Pakistan. The maize roots along with adhered soil were dipped in sterilized distilled water under aseptic conditions, and the resulting soil suspension was used for isolation of rhizobacterial isolates through the serial dilution method. Nutrient agar (HiMedia Laboratories, India) media amended with 50 mM manganese oxide (MnO_2_; Alfa Aesar, United States) was used for the isolation of Mn-resistant rhizobacteria. The serially diluted soil suspension was spread on nutrient agar plates by a glass spreader and inoculated at 30 ± 1°C for 48 h. The resulting fast-growing colonies with distinct morphological appearances were purified through the streak plate method and preserved at −20°C in 50% glycerol stock.

### Manganese-Solubilization Assay

The Mn-tolerant isolates were screened for Mn solubilization by using nutrient agar media modified with MnO_2_ (50 mM) (Sanket et al., 2017). The freshly grown isolates were spot inoculated on Mn-amended nutrient agar and plates were incubated at 30 ± 1°C for 72 h to observe the lightening of the dark brown color around the colonies. After incubation, the plates were flooded with iodine solution as an indicator to record clear zone diameter. The Mn-solubilizing diameter and bacterial growth diameter were taken using a meter rod on a mm scale, with five readings taken from each replication and averaged. The degree of Mn solubilization was determined by measuring the clearing halo zone around the colonies, and the Mn-solubilization index (MSI) and Mn-solubilization efficiency (MSE) were calculated using Formulas 1 and 2, respectively. Such rhizobacterial isolates were termed MSB strains and were selected to be evaluated for their multifunctional PGP characterization.


(1) $$M\,n\,s\,o\,l\,u\,b\,i\,l\,i\,z\,a\,t\,i\,o\,n\,i\,n\,d\,e\,x\,\left(M\,S\,I\right)=\frac{M\,n\,s\,o\,l\,u\,b\,i\,l\,i\,z\,a\,t\,i\,o\,n\,d\,i\,a\,m\,e\,t\,e\,r+B\,a\,c\,t\,e\,r\,i\,a\,l\,C\,o\,l\,o\,n\,y\,D\,i\,a\,m\,e\,t\,e\,r}{B\,a\,c\,t\,e\,r\,i\,a\,l\,c\,o\,l\,o\,n\,y\,d\,i\,a\,m\,e\,t\,e\,r}$$

(2) $$M\,n\,S\,o\,l\,u\,b\,i\,l\,i\,z\,a\,t\,i\,o\,n\,E\,f\,f\,i\,c\,i\,e\,n\,c\,y\,\left(M\,S\,E\%\right)=\frac{M\,n\,S\,o\,l\,u\,b\,i\,l\,i\,z\,a\,t\,i\,o\,n\,D\,i\,a\,m\,e\,t\,e\,r}{B\,a\,c\,t\,e\,r\,i\,a\,l\,C\,o\,l\,o\,n\,y\,D\,i\,a\,m\,e\,t\,e\,r}\times 100$$

The Mn-solubilized concentration by MSB strains was quantified by performing a quantitative Mn solubilization assay. Nutrient broth (HiMedia Laboratories, India) amended with 50 mM MnO_2_ was inoculated with freshly grown strains and incubated with shaking (100 rpm) at 30 ± 1°C for 48 h. For comparison, uninoculated control nutrient broth amended with MnO_2_ was also maintained simultaneously. After incubation, broth cultures were filtered with sterile Whatman No. 01 filter paper and wet digested to determine Mn concentration by following the method of Horwitz (2010). A measured volume of culture filtrate along with HNO_3_ (2 mL) and HCl (10 mL) was taken in a conical flask and covered with a ribbed watch glass. The solution was placed on a hot plate and heated at 350°C to reduce the volume to 25 mL. Then digest was cooled, filtered, and diluted to 100 mL. The filtered digest was read using inductively coupled plasma optical emission spectroscopy (ICP-OES; Optima 7000DV, Perkin Elmer, United States) from the commercial service of Fish Quality Control Labs, Fisheries Research and Training Institute, Manawan, Lahore, Pakistan. The solubilized Mn concentration was determined by drawing calibration of Mn standard solutions of 0, 50, 100, 150, and 200 μg L^–1^. To confirm the results of qualitative and quantitative Mn-solubilization, both assays were repeated twice in triplicate.

### Mineral Solubilization Assays

Manganese-solubilizing strains were screened to evaluate their ability to solubilize P, K, and Zn using insoluble sources. These nutrient solubilization assays were performed by conducting qualitative and quantitative tests and repeated twice in triplicate. For P solubilization, freshly grown Mn-solubilizing strains were spot-inoculated on modified Pikovskaya (PVK) agar media (Pikovskaya, 1948) and incubated at 30 ± 1°C for 72 h. After incubation, the appearance of solubilization zones around spot inoculation was observed and measured through a meter rod on a mm scale. The P solubilization index (PSI) was calculated using the solubilization zone and bacterial growth diameter as reported by Ahmad et al. (2019). The P solubilization efficiency (PSE) was determined by multiplying the ratio of P-solubilizing diameter and bacterial colony diameter with factor of 100 (Ahmad et al., 2019). The P-solubilized concentration was quantified by inoculating Mn-solubilizing strains in PVK broth and incubated in a shaking incubator at 100 rpm and 30 ± 1°C for 72 h. After incubation, broth culture was centrifuged, and culture filtrate (10 mL) was taken in a 100 mL volumetric flask. Ammonium vanadate-molybdate reagent (10 mL) was added to a volumetric flask containing culture filtrate and diluted with distilled water up to the mark on the flask. A blank containing 10 mL ammonium vanadate-molybdate reagent was also added along with samples. The solutions were read through a spectrophotometer (Model G6860A, Agilent Technologies Cary 60 UV-Vis, Australia) at 420 nm (Ryan et al., 2001).

The MSB strains were screened for qualitative Zn solubilization by using Bunt and Rovira agar media amended with 1.24 g L^–1^ bulk insoluble zinc oxide (ZnO) powder (0.1% Zn) (Bunt and Rovira, 1955; Mumtaz et al., 2017, 2019). The MSB strains were spot inoculated for 7 days at 30 ± 1°C, and the Zn-solubilization zone was observed visually. The Zn solubilization index (ZSI) and Zn solubilization efficiency (ZSE) of MSB strains were calculated by measuring the Zn solubilization diameter and bacterial growth diameter by using the formula reported by Ahmad et al. (2019). A quantitative Zn solubilization assay was also performed by inoculating freshly grown MSB strains in Bunt and Rovira broth medium amended with 1.24 g L^–1^ ZnO and incubated at 30 ± 1°C for 7 days under 100 rpm shaking. After incubation, the bacterial culture was filtered and wet digested by following the method of Horwitz (2010). The filtered digest was read using ICP-OES, and the Zn concentration was estimated by drawing a calibration curve. The MSB strains were also screened for K solubilization using the spot inoculation method on modified Aleksandrov agar media amended with insoluble mouse powder (3.0 g L^–1^) (Parmar and Sindhu, 2013). After 7 days of incubation at 30 ± 1°C, agar media was flooded with an iodine solution to observe the visual solubilization zone. The K solubilization zone and bacterial growth diameter were measured, and the K solubilization index (KSI) and K solubilization efficiency (KSE) were calculated as reported by Setiawati and Mutmainnah (2016).

### *In vitro* Screening for Plant Growth-Promoting Characteristics

The MSB strains were characterized for indole-3-acetic acid (IAA) production in the presence of L-tryptophan by following the method of Bric et al. (1991). Strains with an optical density of 0.5 at 600 nm were inoculated in Dworkin and Foster (DF) minimal salt broth amended with 1.0 g L^–1^ L-tryptophan and incubated at 30 ± 1°C for 48 h. After incubation, bacterial culture was filtered and Salkowski reagent was added. Then, the OD of a mixer at 600 nm was recorded through a spectrophotometer, and the auxin concentration was determined by plotting the standard curve of the IAA standard solution. Siderophore production by MSB strains was determined by using a blue agar chrome azurol S (CAS) assay as reported by Louden et al. (2011). The strains were spot inoculated on blue agar CAS medium and incubated at 30 ± 1°C for 48 h. The production of orange halo zones around bacterial growth was observed and was considered siderophore positive. For ammonia (NH_3_) production, strains were inoculated in peptone broth and incubated at 30 ± 1°C for 48 h. After incubation, culture filtrates were treated with Nessler’s reagent, the production of yellow to brown color was observed (Cappuccino and Welsh, 2017), and OD at 530 nm was recorded through a spectrophotometer (Agilent Technologies, Australia). The extracellular enzymes *viz*. catalase, oxidase, amylase, protease, and lipase activities were determined by following the standard method of Cappuccino and Welsh (2017). The cell and colony morphology of MSB strains were also determined by following the protocol reported by Breakwell et al. (2007).

### Screening for Maize Growth Promotion

A sand culture pot trial was conducted to screen the ability of the MSB strain to promote maize growth and Mn uptake in the presence of an insoluble Mn source in a sterilized pure sand culture. Pure sieved (2 mm) sand was amended with 50 mM MnO_2_ and used to fill plastic pots (length 11 cm; top diameter 11 cm; and bottom diameter 6 cm). These pots were moistened with half-strength Mn-deficient Hoagland solution and autoclaved. To prevent oxidation, ferrous sulfate solution in water was autoclaved separately in a headspace of nitrogen in a tube with a fixed stopper and mixed with other contents of Hoagland solution after autoclaving. The MSB strains were cultured in DF minimal salt broth at 30 ± 1°C in a shaking incubator for 48 h. Maize seeds of variety Afghoi SG-2002 were disinfected by dipping in 95% ethanol for 5 min and then dipped in 0.2% mercury chloride (HgCl_2_) solution for 3 min. Seeds were then washed several times with sterile distilled water. The surface-sterilized maize seeds were inoculated with bacterial cultures for 30 min. A total of six seeds were sown in each plastic pot and upon germination of maize, three maize seedling were maintained in each pot. For the control treatment, maize seeds were sown after soaking in DF minimal salt broth without bacterial inoculum. The pots were arranged in completely randomized design having three replications in the growth room under optimum conditions of 28 ± 2°C temperature along with 16 h of light and 8 h of a dark period. The pots were irrigated with an equal volume of half-strength Mn-deficient Hoagland solution. After 3 weeks of incubation, harvesting was performed, and growth parameters of plants, including shoot and root length, shoot fresh and dry biomass, and root fresh and dry biomass were recorded by following the standard methods reported by Mumtaz et al. (2017). Maize shoot and root samples were oven-dried and ground into a powder. One gram of maize shoot and root were wet digested by following the method reported in the above section of Mn concentration determination. The digested sample was diluted up to 50 mL and filtered. The digested samples were read using ICP-OES and the Mn concentration was calculated after plotting the calibration curve from the Mn standard solution (Horwitz, 2010).

### Identification of Selected Manganese-Solubilizing Bacterial Strains

The MSB strains ASH6, ASH11, ASH19, ASH20, and ASH22 were selected based on their minerals solubilization and maize growth-promoting ability. The method of Cheneby et al. (2004) was adopted to extract the genomic DNA of selected strains through proteinase K treatment. The genomic DNA (2.5 μL) was amplified through a thermocycler (Eppendorf, United States) using universal primers targeting the 16S rRNA gene following the program reported by Mumtaz et al. (2017). The amplified PCR product was confirmed on a 1% agarose gel and subjected to sequencing from the commercial service of Macrogen (Seoul, South Korea). The resulting sequences were blasted using the MegaBlast service on NCBI servers. Closely matched sequences of strains were retrieved from a database, and a neighbor-joining phylogenetic tree was created using MEGA 7.0.14 software (Kumar et al., 2016). The sequences were submitted to the NCBI gene bank, and accession numbers of the MSB strains were obtained.

### Pot Experiment

A soil culture pot experiment was performed to evaluate the effect of selected MSB strains *viz*. ASH6, ASH11, ASH19, ASH20, and ASH22 inoculation on maize growth and nutrient uptake. Bacterial cultures were prepared by growing them in nutrient broth amended with 50 mM MnO_2_ in a shaking incubator at 30 ± 1°C and 100 rpm for 48 h of incubation. Maize seeds of the Afghoi SG-2002 variety were soaked in bacterial inoculum for 30 min. The uninoculated control treatment was prepared by soaking maize seeds in a similar MnO_2_-amended nutrient broth but without bacterial inoculation. A total of six maize seeds inoculated with respective bacterial strains were sown in each pot and three maize seedling after germination were maintained for the experiment. This pot trial was conducted in a greenhouse of IMBB, UOL, Pakistan, located at Latitude: 31.39N, Longitude: 74.24E, and 206 meters elevation above sea level under natural climatic conditions. The soil was collected from farmer fields and analyzed for physicochemical characteristics (Ryan et al., 2001). The soil used for the experiment was sandy loam texture, 8.1 pH, 0.43 dS m^–1^ electrical conductivity, 0.30% organic matter, 0.02% total N, 5.5 mg kg^–1^ available P, 175 mg kg^–1^ extractable K, 14.36 mg kg^–1^ extractable Mn, and 26.90 mg kg^–1^ total Mn concentration. Pots 30.5 cm in width were filled with 10 kg of sieved soil, and maize seeds were sown. The pots were arranged in completely randomized design, and treatments were replicated in triplicate. The plants were fertilized with two equal doses of 0.54 g of N in the form of urea and applied at the V2 and anthesis stages. The P (0.81 g) and K (0.54 g) were applied in each pot in the form of diammonium phosphate and sulfate of potash, respectively, at the time of sowing. Pots were irrigated with good-quality tap water, and thinning was performed after seed germination. At the cob formation stage, the chlorophyll contents of the second top leaf were recorded through a SPAD chlorophyll meter (Hansatech Instruments, England). At physiological maturity, the growth attributes, *viz*., plant height, root length, shoot dry weight, and root dry weight were recorded. Maize root and shoot samples were wet digested as reported in the above sections (Horwitz, 2010) and analyzed for N, P, K, and Mn concentrations. The plant digested samples were analyzed for the determination of total N through the Kjeldahl method, P concentration through a colorimetric method, and K concentration through a flame photometer (BWB Technologies, United Kingdom; Jackson, 1962). The plant digested samples were also analyzed for Mn concentration using ICP-OES as reported in the above section.

### Statistical Analysis

A statistical method was employed to characterize and predict the ability of the MSB strain to promote maize growth. The MSB strain characterization and pot trial data were subjected to one-way analysis of variance (ANOVA) by employing a model completely randomized design with the computer software Statistix v. 8.1. The standard error of three replications in each MSB treatment was calculated by dividing the standard deviation by the square root of three by using Microsoft Excel 2019. The treatment means were compared through the least significant difference (LSD) test at a 5% probability level (Steel et al., 1997). The pairwise Pearson correlation analysis between the nutrient concentration in roots and shoots of maize in soil culture pot trial was performed using computer software Origin Pro 2021 (OriginLab Corporation, Northampton, MA, United States).

## Results

### Isolation of Manganese-Solubilizing Bacterial Strains

**FIGURE 1:**
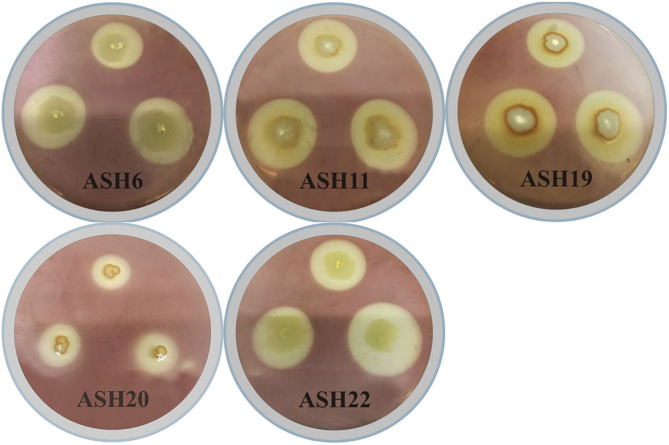
Solubilization of manganese oxide (MnO_2_) by manganese solubilizing bacterial (MSB) strains *viz*. ASH6, ASH11, ASH19, ASH20, and ASH22 on MnO_2_-amended nutrient agar medium for 48 h; After incubation, dilute iodine solution was flooded on agar medium and appearance of manganese solubilization zone around bacterial growth was considered positive for solubilization of MnO_2_.

In the present study, a total of 50 Mn-tolerant isolates were isolated from the maize rhizosphere and were coded A1, A2, A3 …A50. These isolates were screened for Mn solubilization by growing on nutrient agar amended with insoluble MnO_2_. Thirteen Mn-tolerant isolates showing solubilization of Mn were coded as ASH4, ASH6, ASH7, ASH8, ASH9, ASH10, ASH11, ASH12, ASH17, ASH19, ASH20, ASH22, and ASH24. The qualitative Mn solubilization ability of these strains is presented in Table 1 and Figure 1. *In vitro* screening of selected strains for MnO_2_ solubilization showed that the Mn solubilization diameter ranged from 7.5 ± 0.03 mm to 35.5 ± 1.06 mm, while the bacterial growth diameter ranged from 7.0 ± 0.01 mm to 19.0 ± 0.70 mm. The maximum Mn solubilization diameter and bacterial growth diameter were reported by strain ASH22. Strain ASH20 showed a comparatively lower growth diameter; however, strain ASH20 showed a better solubilization diameter after strain ASH22 and was non-significant to strain ASH10. The lowest Mn solubilization diameter was obtained with strain ASH9.

**TABLE 1:** Qualitative and quantitative solubilization of manganese by rhizobacterial strains isolated from maize rhizosphere.

Strains	MSD (mm)	BGD (mm)	MSI	MSE (%)	SMC (μg mL^–1^)
Control	ND	ND	ND	ND	3.42 ± 0.11^e^
ASH4	26.00 ± 0.70^c^	10.50 ± 0.35^b−e^	3.47 ± 0.01^b^	247.73 ± 1.60^b^	3.59 ± 0.02^e^
ASH6	25.54 ± 0.35^cd^	14.00 ± 1.41^b^	2.86 ± 0.07^b^	186.46 ± 21.36^b^	10.73 ± 0.59^a^
ASH7	9.50 ± 0.03^g^	8.45 ± 0.03^cde^	2.12 ± 0.03^b^	111.80 ± 0.49^b^	3.66 ± 0.12^e^
ASH8	8.05 ± 0.01^g^	7.02 ± 0.01^e^	2.14 ± 0.01^b^	114.30 ± 0.01^b^	3.80 ± 0.17^e^
ASH9	7.50 ± 0.03^g^	6.50 ± 0.03^e^	2.15 ± 0.02^b^	115.50 ± 0.84^b^	3.69 ± 0.04^e^
ASH10	29.50 ± 0.35^b^	12.21 ± 1.41^bcd^	3.52 ± 0.12^b^	252.14 ± 26.76^b^	3.79 ± 0.14^e^
ASH11	12.45 ± 0.03^f^	11.00 ± 0.02^b−e^	2.14 ± 0.03^b^	113.60 ± 3.21^b^	6.58 ± 0.27^b^
ASH12	26.00 ± 0.01^c^	13.00 ± 0.21^bc^	3.11 ± 0.13^b^	211.73 ± 34.47^b^	3.80 ± 0.02^e^
ASH17	23.55 ± 1.06^d^	13.50 ± 0.35^b^	2.74 ± 0.06^b^	173.90 ± 3.30^b^	3.56 ± 0.13^e^
ASH19	14.03 ± 0.01^ef^	12.00 ± 0.01^bc^	2.17 ± 0.02^b^	116.70 ± 0.02^b^	4.89 ± 0.06^c^
ASH20	29.00 ± 0.70^b^	7.50 ± 2.47^de^	5.86 ± 1.26^a^	486.36 ± 151.06^a^	10.08 ± 0.35^a^
ASH22	35.51 ± 1.06^a^	19.00 ± 0.70^a^	2.87 ± 0.05^b^	186.94 ± 1.37^b^	5.87 ± 0.13^bc^
ASH24	16.01 ± 0.01^e^	9.50 ± 1.06^b−e^	2.73 ± 0.08^b^	172.73 ± 19.28^b^	3.59 ± 0.12^e^
LSD	1.09	2.27	0.89	88.72	0.31

*The strains showed qualitative and quantitative solubilization of manganese (Mn) and were termed Mn-solubilizing bacterial (MSB) strains; qualitative Mn solubilization by MSB strains was determined by spot inoculating bacterial biomass on MnO_2_-amended nutrient agar media and Mn solubilization diameter (MSD) was recorded after 48 h of incubation; Mn solubilization index (MSI) and Mn solubilization efficiency (MSE) were calculated by using MSD and bacterial growth diameter (BGD); quantitative Mn solubilization was estimated through inoculating the strains in MnO_2_-amended nutrient broth media and solubilized Mn concentration (SMC) was detected through inductively coupled plasma optical emission spectroscopy; data presented are the mean of three replications ± standard error and least significant difference (LSD) test was performed at 5% (*p* ≤ 0.05) probability level; the means in a vertical line for each attributes sharing common letters were considered statistically similar to each other; ND, not detected.*

The data regarding MSI and MSE calculated through Mn solubilization diameter and bacterial growth diameter showed that the most efficient strain was ASH20 which reported 5.86 ± 1.26 MSI and 486.4 ± 151.06% MSE (Table 1). The strains ASH4, ASH10, and ASH12 also showed better MSI and MSE and were non-significant to each other and other tested strains except strain ASH20. The strains ASH7, ASH8, and ASH9 showed the lowest MSI and MSE. The strains ASH6 (10.73 ± 0.59 μg mL^–1^) and ASH20 (10.08 ± 0.35 μg mL^–1^) reported maximum Mn solubilized concentration and were non-significant to each other. Following these strains, ASH11 (6.58 ± 0.27 μg mL^–1^) and ASH22 (5.87 ± 0.13 μg mL^–1^) also showed better Mn solubilized concentrations and were non-significant to each other. The control broth showed a Mn concentration of 3.42 ± 0.11 μg mL^–1^ and was non-significant with the solubilized Mn concentration by strains ASH4, ASH7, ASH8, ASH9, ASH12, ASH17, and ASH24.

### *In vitro* Mineral Solubilization by Manganese-Solubilizing Bacterial Strains

**FIGURE 2:**
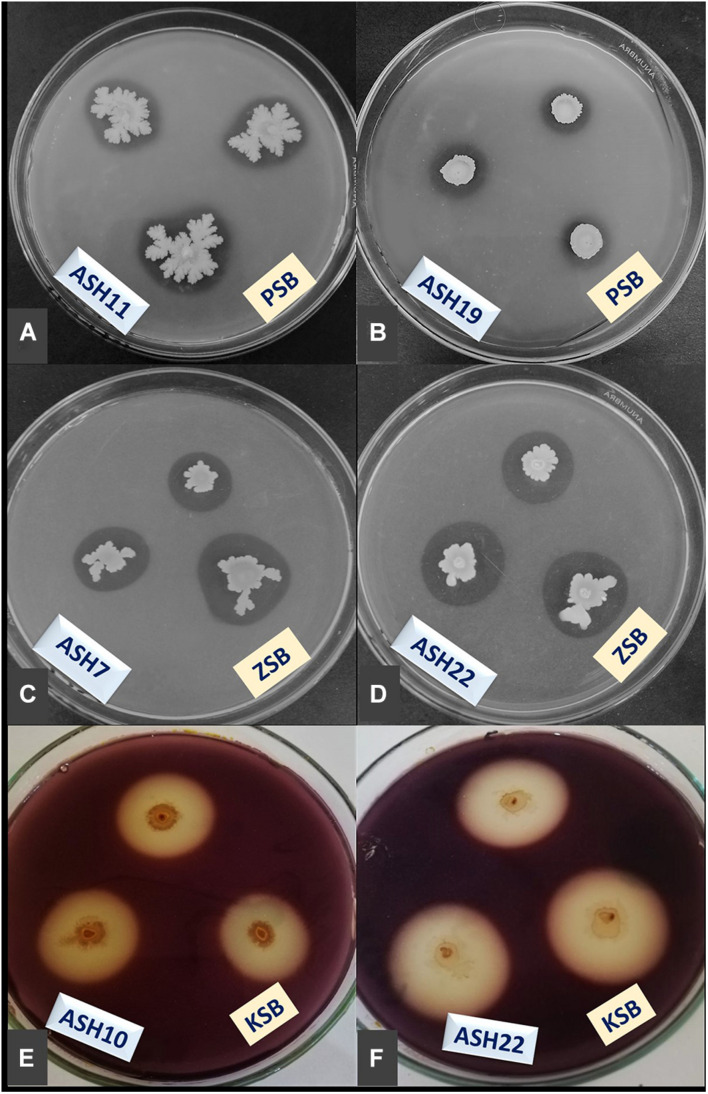
Demonstration of minerals solubilization; phosphorus (P) by ASH11 **(A)** and ASH19 **(B)**, zinc (Zn) by ASH7 **(C)** and ASH22 **(D)**, and potassium (K) by ASH10 **(E)** and ASH22 **(F)**; manganese solubilizing bacterial (MSB) strains were spot inoculated on Pikovskaya agar amended with tri-calcium phosphate and tris-minimal salt agar amended with zinc oxide for P and Zn solubilization; K solubilization was determined by inoculating MSB strains on mica-amended Aleksandrov agar media; these solubilization assays were incubated for 7 days and appearance of solubilization zones were considered positive for respective mineral solubilization; K solubilization zones were observed through diluting agar media with dilute iodine solution for 10 min; PSB, P solubilizing bacteria; ZSB, Zn solubilizing bacteria; KSB, K solubilizing bacteria.

A qualitative P solubilization assay revealed that all the MSB strains were positive for P solubilization (Table 2 and Figure 2). The maximum P solubilization diameter (25.7 ± 0.72 mm) and bacterial growth diameter (21.7 ± 0.72 mm) were shown by strain ASH17. The strains ASH12, ASH19, and ASH20 also showed better P solubilization diameters and bacterial growth diameters and were non-significant to each other but significant to the other tested strains. The minimum P solubilization diameter (10.0 ± 0.09 mm) and bacterial growth diameter (4.3 ± 0.27 mm) were reported by strain ASH6. Strains ASH6, ASH8, and ASH11 showed maximum PSIs (3.33 ± 0.10, 3.11 ± 0.07, and 3.28 ± 0.11, respectively) and PSEs (232.9 ± 13.61, 211.0 ± 9.07, and 227.8 ± 12.02, respectively) and were non-significant to each other but significant from the other tested strains. Quantitative P solubilization revealed that the maximum solubilized P concentration was obtained from strain ASH12 (6.67 ± 0.69 μg mL^–1^), followed by strain ASH22 (4.26 ± 0.07 μg mL^–1^). The uninoculated control broth reported a P concentration of 0.43 ± 0.02 μg mL^–1^ (Table 2).

**TABLE 2:** Qualitative and quantitative solubilization of phosphorus by manganese solubilizing bacterial strains isolated from maize rhizosphere.

Strains	PSD (mm)	BGD (mm)	PSI	PSE (%)	SPC (μg mL^–1^)
Control	ND	ND	ND	ND	0.43 ± 0.02^*e*^
ASH4	13.67 ± 0.27^de^	9.33 ± 0.27^f^	2.47 ± 0.03^c^	147.16 ± 3.78^c^	1.67 ± 0.12^cd^
ASH6	10.00 ± 0.09^f^	4.33 ± 0.27^g^	3.33 ± 0.10^a^	232.93 ± 13.61^a^	2.27 ± 0.24^c^
ASH7	11.33 ± 0.54^ef^	6.33 ± 0.54^g^	2.80 ± 0.02^b^	180.95 ± 7.77^b^	1.93 ± 0.26^cd^
ASH8	14.00 ± 0.94^d^	6.65 ± 0.54^g^	3.11 ± 0.07^a^	211.11 ± 9.07^a^	2.50 ± 0.79^c^
ASH9	18.66 ± 0.27^c^	12.67 ± 0.27^de^	2.47 ± 0.01^c^	147.44 ± 1.04^c^	1.80 ± 0.32^cd^
ASH10	12.10 ± 0.47^def^	6.65 ± 0.27^g^	2.82 ± 0.12^b^	181.75 ± 14.64^b^	0.63 ± 0.05^d^
ASH11	11.33 ± 1.08^ef^	5.00 ± 0.47^g^	3.28 ± 0.11^a^	227.78 ± 12.02^a^	1.83 ± 0.21^cd^
ASH12	23.00 ± 0.81^b^	15.66 ± 1.18^bc^	2.48 ± 0.05^c^	148.29 ± 6.68^c^	6.67 ± 0.69^a^
ASH17	25.67 ± 0.72^a^	21.67 ± 0.72^a^	2.19 ± 0.07^d^	118.12 ± 0.63^d^	2.50 ± 0.41^c^
ASH19	22.33 ± 1.18^b^	18.00 ± 0.94^b^	2.24 ± 0.09^cd^	124.07 ± 0.75^cd^	1.83 ± 0.59^cd^
ASH20	21.67 ± 0.72^b^	17.33 ± 0.98^bc^	2.25 ± 0.06^cd^	125.54 ± 3.19^cd^	0.73 ± 0.09^d^
ASH22	18.00 ± 0.47^c^	15.00 ± 0.81^cd^	2.21 ± 0.04^cd^	120.07 ± 4.37^cd^	4.26 ± 0.07^b^
ASH24	14.00 ± 0.47^d^	11.33 ± 0.54^ef^	2.24 ± 0.03^cd^	122.89 ± 3.17^cd^	0.80 ± 0.08^d^
LSD	1.21	1.16	0.14	13.48	0.67

*Manganese solubilizing bacterial (MSB) strains were screened for qualitative solubilization of phosphorus (P) by spot inoculating bacterial biomass on Pikovskaya (PVK) agar media; P solubilization diameter (PSD) and bacterial growth diameter (BGD) were recorded after 7 days of incubation; the P solubilization index (PSI) and P solubilization efficiency (PSE) were calculated through using PSD and BGD; the quantitative P solubilization was determined by inoculating PVK broth for 7 days and solubilized P concentration (SPC) was detected through a colorimetric method by using a spectrophotometer; data presented are the mean of three replications ± standard error and least significant difference (LSD) test was performed at 5% (*p* ≤ 0.05) probability level; the means in a vertical line for each attributes sharing common letters were considered statistically similar to each other; ND, not detected.*

A qualitative assay for Zn solubilization revealed that all the tested bacterial strains were positive for Zn solubilization and data are given in Table 3 and Figure 2. Strain ASH4 a reported maximum growth diameter of 19.0 ± 2.12 mm and Zn solubilization diameter of 28.5 ± 1.76 mm. Strains ASH7, ASH10, and ASH20 also showed better Zn solubilization diameters of 22.0 ± 1.41 mm, 20.0 ± 0.65 mm, and 22.2 ± 0.35 mm, respectively, and were non-significant to each other; however, they were significant to the other tested strains. For ZSI and ZSE values, ASH19 was the leading strain having maximum values of 4.73 ± 0.33 for ZSI and 372.3 ± 37.12% for ZSE followed by strain ASH8, having 4.63 ± 0.08 for ZSI and 361.8 ± 8.83% for ZSE. The better qualitative Zn solubilizing strains ASH6, ASH11, ASH19, ASH20, and ASH22 were tested in a quantitative assay for solubilization of Zn, and data are presented in Table 3. The uninoculated control was composed of 0.12 ± 0.01 μg mL^–1^ Zn. Strain ASH19 reported a maximum solubilized Zn concentration of 23.17 ± 0.21 μg mL^–1^, followed by strain ASH22, which solubilized Zn concentrations up to 22.05 ± 0.19 μg mL^–1^. The MSB strains were also screened for qualitative K solubilization, and the data are reported in Table 4 and Figure 2. The results revealed that six bacterial strains (ASH4, ASH10, ASH12, ASH17, ASH20, and ASH22) out of 13 tested MSB strains showed K solubilization. Strain ASH22 gave a maximum K solubilization diameter of 25.5 ± 1.76 mm having bacterial growth diameter of 3.5 ± 0.35 mm followed by ASH4 which reported 25 ± 1.41 mm of K solubilization diameter and 5.0 ± 0.70 mm of bacterial growth diameter. Strain ASH22 reported a maximum KSI of 8.33 ± 0.20 and KSE of 733.2 ± 23.57%, followed by strain ASH12, which had a KSI of 8.04 ± 0.87 and KSE of 704.4 ± 91.33%. Both of these strains were non-significant for ASH4 and ASH10 (Table 4).

**TABLE 3:** Qualitative and quantitative solubilization of zinc by manganese solubilizing bacterial strains isolated from maize rhizosphere.

Strains	ZSD (mm)	BGD (mm)	ZSI	ZSE (%)	SZC (μg mL^–1^)
Control	ND	ND	ND	ND	0.12 ± 0.01^f^
ASH4	28.50 ± 1.76^a^	19.00 ± 2.12^a^	3.52 ± 0.13^cde^	151.37 ± 7.63^cde^	NT
ASH6	18.50 ± 0.03^cde^	10.00 ± 0.70^bc^	2.86 ± 0.02^cd^	185.86 ± 9.64^cd^	16.54 ± 0.14^d^
ASH7	22.00 ± 1.41^bc^	11.50 ± 1.06^bc^	2.92 ± 0.05^c^	192.13 ± 5.43^c^	NT
ASH8	14.50 ± 0.35^fgh^	4.00 ± 0.01^e^	4.63 ± 0.08^ab^	361.80 ± 8.83^ab^	NT
ASH9	13.00 ± 1.41^gh^	9.00 ± 0.70^cd^	2.44 ± 0.11^cde^	143.15 ± 4.41^cde^	NT
ASH10	20.00 ± 0.65^bcd^	12.00 ± 0.02^bc^	2.67 ± 0.40^cde^	166.36 ± 0.02^cde^	NT
ASH11	12.50 ± 0.35^h^	4.00 ± 0.01^e^	4.13 ± 0.08^b^	311.90 ± 8.83^b^	17.86 ± 0.25^c^
ASH12	16.00 ± 0.01^e–h^	12.50 ± 0.35^bc^	2.28 ± 0.06^e^	128.02 ± 3.62^e^	NT
ASH17	17.45 ± 0.35^def^	13.00 ± 0.70^b^	2.35 ± 0.02^de^	135.42 ± 4.62^de^	NT
ASH19	16.50 ± 0.35^d–g^	4.50 ± 0.35^e^	4.73 ± 0.33^a^	372.30 ± 37.12^a^	23.17 ± 0.21^a^
ASH20	22.20 ± 0.35^b^	12.00 ± 0.70^bc^	2.88 ± 0.01^cd^	188.14 ± 8.15^cd^	12.20 ± 0.31^e^
ASH22	8.50 ± 0.35^i^	6.00 ± 0.04^de^	2.42 ± 0.05^cde^	141.07 ± 5.89^cde^	22.05 ± 0.19^b^
LSD	1.63	1.62	0.25	24.97	0.42

*Manganese solubilizing bacterial (MSB) strains were screened for qualitative solubilization of zinc (Zn) by spot inoculating bacterial biomass on ZnO_2_-amended tris-minimal salt agar media. Zn solubilization diameter (ZSD) and bacterial growth diameter (BGD) were recorded after 7 days of incubation; the Zn solubilization index (ZSI) and Zn solubilization efficiency (ZSE) were calculated through using ZSD and BGD; the quantitative Zn solubilization was determined by inoculating the ZnO_2_-amended tris-minimal salt broth for 7 days and solubilized Zn concentration (SZC) was detected through inductively coupled plasma optical emission spectroscopy; data presented here are the mean of three replications ± standard error and least significant difference (LSD) test was performed at 5% (*p* ≤ 0.05) probability level; the means in a vertical line for each attributes sharing common letters were considered statistically similar to each other; NT, not tested.*

**TABLE 4:** Qualitative solubilization of potassium by manganese solubilizing bacterial strains isolated from maize rhizosphere.

Strains	KSD (mm)	BGD (mm)	KSI	KSE (%)
ASH4	25.00 ± 1.41^a^	5.00 ± 0.70^b^	6.13 ± 0.37^ab^	512.50 ± 44.19^ab^
ASH6	ND	ND	ND	ND
ASH7	ND	ND	ND	ND
ASH8	ND	ND	ND	ND
ASH9	ND	ND	ND	ND
ASH10	23.50 ± 1.06^a^	5.00 ± 0.70^b^	5.96 ± 0.84^ab^	495.83 ± 91.33^ab^
ASH11	ND	ND	ND	ND
ASH12	24.00 ± 0.70^a^	3.50 ± 0.35^b^	8.04 ± 0.87^a^	704.17 ± 91.33^a^
ASH17	22.00 ± 1.41^a^	8.00 ± 0.70^a^	3.76 ± 0.02^bc^	276.19 ± 6.73^bc^
ASH19	ND	ND	ND	ND
ASH20	8.00 ± 0.01^b^	4.50 ± 0.35^b^	2.80 ± 0.10^c^	180.00 ± 14.14^c^
ASH22	25.50 ± 1.76^a^	3.50 ± 0.35^b^	8.33 ± 0.20^a^	733.33 ± 23.57^a^
ASH24	ND	ND	ND	ND
LSD	2.42	1.12	1.14	113.84

*Manganese solubilizing bacterial strains (MSB); the MSB strains were screened for qualitative solubilization of potassium (K) by spot inoculating bacterial biomass on mica-amended Aleksandrov agar media for 7 days and K solubilization diameter (KSD) and bacterial growth diameter (BGD) were recorded; the K solubilization index (KSI) and K solubilization efficiency (KSE) were calculated through using KSD and BGD; data presented here are the mean of three replications ± standard error and least significant difference (LSD) test was performed at 5% (*p* ≤ 0.05) probability level; the means in a vertical line for each attributes sharing common letters were considered statistically similar to each other; ND, not detected.*

### *In vitro* Characterization of Manganese-Solubilizing Bacterial Strains

The MSB strains were evaluated *in vitro* for PGP characteristics and enzymatic activities, and findings are depicted in Table 5. The MSB strains were tested for IAA production in the presence of L-tryptophan, and the results revealed that eight out of thirteen strains were able to produce IAA. The maximum IAA of 24.76 ± 0.42 μg mL^–1^ was produced by strain ASH17 followed by strain ASH10 which had 23.13 ± 1.73 μg mL^–1^ IAA. Both of these strains were non-significant to each other but significantly different from the other tested strains. The strains ASH11 (19.99 ± 0.28 μg mL^–1^) and ASH19 (18.73 ± 0.12 μg mL^–1^) also showed significantly better IAA production and were statistically similar to each other. NH_3_ production in terms of OD revealed that strains ASH4 and ASH10 were statistically similar to each and reported maximum ODs of 1.57 ± 0.15 and 1.36 ± 0.06, respectively. Strains ASH9 and ASH11 also showed better NH_3_ production up to 1.16 ± 0.04 and 1.11 ± 0.08, respectively, while the uninoculated control reported minimum NH_3_ production (0.46 ± 0.04). Siderophore production was recorded in terms of the appearance of the halo zone around bacterial growth on CAS agar medium as shown in Supplementary Figure 1. Eight out of thirteen MSB strains were positive for siderophore production (Supplementary Figure 2). The amylase, lipase, and protease activities were determined by inoculating strains on respective agar media, and the results were observed in terms of the appearance of a halo zone around bacterial growth, as shown in Supplementary Figure 3. All the tested strains were positive for protease activity except strain ASH24, while strains ASH7, ASH8, and ASH19 were unable to perform amylase activity. The majority of MSB strains were also positive for lipase activity, while, strains ASH6, ASH9, and ASH12 were negative for catalase activity. All the strains were also positive for oxidase activity except strains ASH6, ASH9, and ASH19. A bacterial morphological chart was made by observing different morphological characteristics in terms of color, texture, margin, elevation, and form (Supplementary Table 1). Morphological observation revealed that most of the tested strain colonies were of white to yellow in color, circular in shape, smooth in appearance with raised elevation and entire margin.

**TABLE 5:** Plant growth-promoting characteristics and enzymatic activities by manganese solubilizing strains isolated from maize rhizosphere.

Strains	IAA Prod. (μg mL^–1^)	NH_3_ Prod.	Siderophores	Catalase activity	Protease activity	Amylase activity	Lipase activity	Oxidase activity
Control	4.92 ± 0.22^f^	0.46 ± 0.04^g^	ND	ND	ND	ND	ND	ND
ASH4	7.86 ± 0.59^e^	1.57 ± 0.15^a^	+ve	+ve	+ve	+ve	+ve	+ve
ASH6	16.99 ± 1.74^c^	0.56 ± 0.05^fg^	−ve	−ve	+ve	+ve	+ve	−ve
ASH7	ND	0.88 ± 0.05^de^	−ve	+ve	+ve	−ve	+ve	+ve
ASH8	ND	0.42 ± 0.03^g^	+ve	+ve	+ve	−ve	+ve	+ve
ASH9	ND	1.16 ± 0.04^bc^	−ve	−ve	+ve	+ve	−ve	−ve
ASH10	23.13 ± 1.73^a^	1.36 ± 0.06^ab^	+ve	+ve	+ve	+ve	−ve	+ve
ASH11	19.99 ± 0.28^b^	1.11 ± 0.08^bcd^	+ve	+ve	+ve	+ve	+ve	+ve
ASH12	ND	0.58 ± 0.08^fg^	+ve	−ve	+ve	+ve	+ve	+ve
ASH17	24.76 ± 0.42^a^	1.11 ± 0.03^cd^	+ve	+ve	+ve	+ve	+ve	+ve
ASH19	18.73 ± 0.12^bc^	0.78 ± 0.02^ef^	−ve	+ve	+ve	−ve	−ve	−ve
ASH20	12.56 ± 0.61^d^	0.41 ± 0.01^g^	+ve	+ve	+ve	+ve	−ve	+ve
ASH22	10.13 ± 0.59^de^	1.08 ± 0.09^cd^	−ve	+ve	+ve	+ve	−ve	+ve
ASH24	ND	1.22 ± 0.08^bc^	+ve	+ve	−ve	+ve	+ve	+ve
LSD	1.31	0.12	ND	ND	ND	ND	ND	ND

*Manganese solubilizing bacterial (MSB) strains were screened for various biochemical characterization responsible for plant growth promotion and enzymatic activities; data presented are the mean of three replications ± standard error; the means in a vertical line for each attributes sharing common letters were considered statistical similar to each other and least significant difference (LSD) test was performed at 5% (*p* ≤ 0.05) probability level. The symbol, +ve represents the presence of the traits while the symbol, −ve shows the absence of the trait; ND, not detected.*

### Promotion of Maize Growth Under the Axenic Condition

**FIGURE 3:**
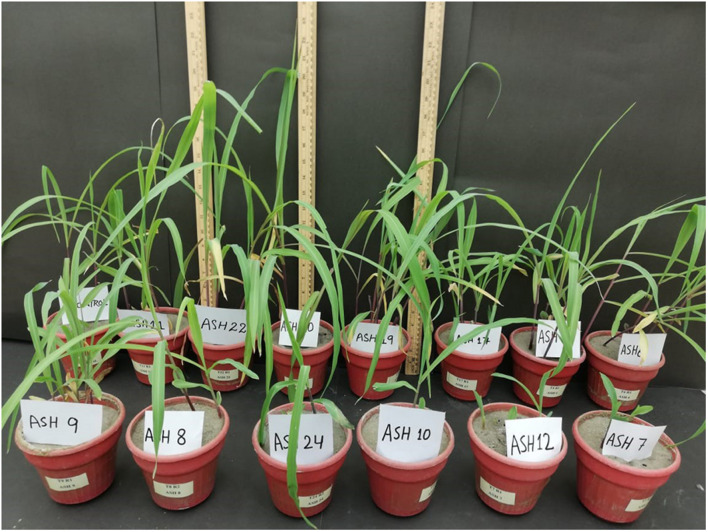
Effect of manganese solubilizing bacterial (MSB) strains to increase maize growth and uptake of manganese (Mn) in shoots and roots; the pot experiment was conducted through sowing MSB strains inoculated maize seeds in pure sand culture amended with manganese oxide (MnO_2_; 50 mM); Maize seedling was grown up to 21 days after germination under 16 h of light and 8 h of darkness by arranging the pots in complete randomized design in triplicate.

**FIGURE 4:**
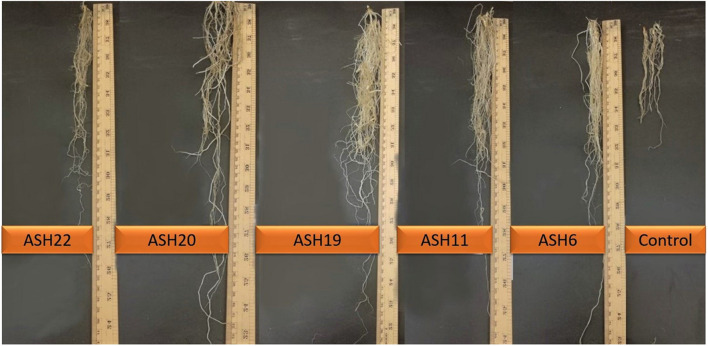
Effect of manganese solubilizing bacterial strains on root length of maize under pure sand culture amended with manganese oxide in pot experimental conditions.

**FIGURE 5:**
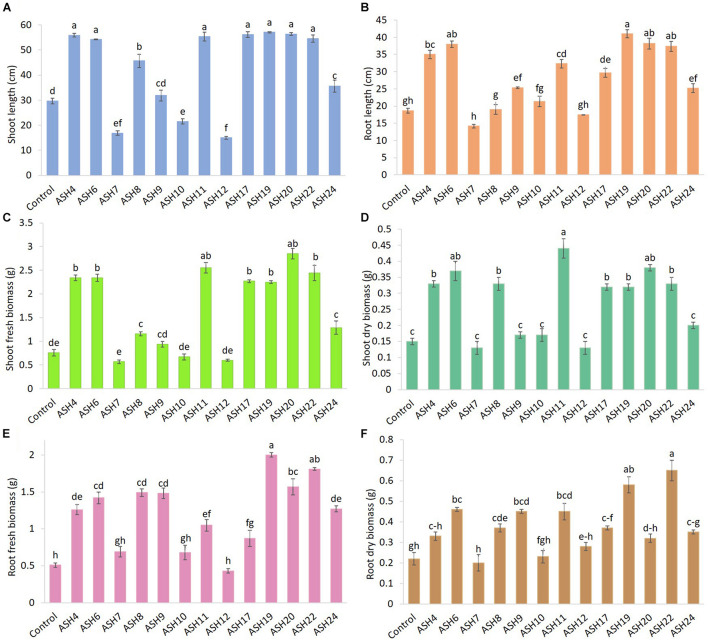
Effect of Mn solubilizing bacterial (MSB) strains on maize growth and uptake of manganese under axenic conditions; the surface-sterilized maize seeds were inoculated with MSB and grown-up to 3 weeks after germination under 16 h of light and 8 h of a dark period of axenic conditions in small pots trials; the data was recorded in terms of shoot length **(A)**, root length **(B)**, shoot fresh biomass **(C)**, shoot dry biomass **(D)**, root fresh biomass **(E)**, and root dry biomass **(F)**; top five best-performing strains ASH6, ASH11, ASH19, ASH20, and ASH22 were selected for identification; the control had no inoculum (uninoculated) and contains only broth; data presented are the mean of three replications ± standard error; the means sharing common letters were considered statistical similar to each other and least significant difference (LSD) test was performed at 5% (*p* ≤ 0.05) probability level.

**FIGURE 6:**
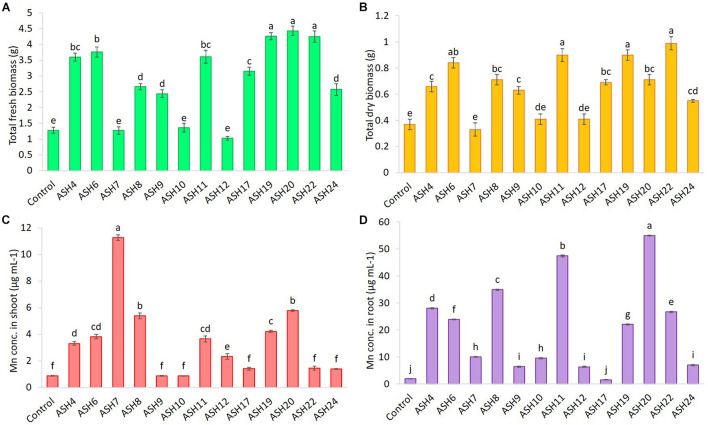
The total fresh biomass **(A)**, total dry biomass **(B)**, uptake of Mn in root **(C)** and shoot **(D)** of maize grown with Mn solubilizing bacterial (MSB) strains; the surface-sterilized maize seeds were inoculated with MSB and grown-up to 3 weeks after germination under 16 h of light and 8 h of a dark period of axenic conditions in small pots trials; the control had no inoculum (uninoculated) and contains only broth; data presented are the mean of three replications ± standard error; the means sharing common letters were considered statistical similar to each other and least significant difference (LSD) test was performed at 5% (*p* ≤ 0.05) probability level.

The MSB strains were screened for their ability to promote maize growth and Mn uptake under axenic conditions in a pure sand culture amended with a MnO_2_ pot trial. The maize seeds inoculated with MSB strains having the ability to solubilize Mn and other minerals significantly promoted maize growth after 21 days of germination (Figures 3, 4). The uninoculated control reported minimum shoot length (29.66 ± 1.18 cm), root length (18.66 ± 0.72 cm), shoot fresh biomass (0.76 ± 0.07 g), root fresh biomass (0.51 ± 0.03 g), shoot dry biomass (0.15 ± 0.01 g), root dry biomass (0.22 ± 0.03 g), total fresh biomass (1.28 ± 0.10 g), and total dry biomass (0.37 ± 0.04 g) (Figures 5, 6). Compared to the uninoculated control, the majority of MSB strains promoted maize growth attributes in the presence of an insoluble source of Mn; however strains ASH7, ASH10, and ASH12 were unable to promote maize growth compared to the uninoculated control and were considered non-PGP MSB strains. The effect of MSB strains on shoot growth is shown in Supplementary Figure 4. The highest shoot length of 57.08 ± 0.24 cm with an increase up to 92% over uninoculated control was shown by strain ASH19. Strain ASH19 was statistically similar to strains ASH4, ASH6, ASH11, ASH17, ASH20, and ASH22, however, these strains were statistically significant compared to other tested strains and the uninoculated control. The shoot lengths due to strains ASH7, ASH10, and ASH12 were 16.83 ± 0.91 cm, 21.50 ± 1.02 cm, and 15.00 ± 0.58 cm, respectively, which were non-significant and/or lower than the shoot length of the uninoculated control (Figure 5A). Figure 4 demonstrates a significant increase in root length of maize in the presence of an insoluble source of Mn, however, strains ASH7, ASH8, ASH10, and ASH12 reported non-significant root lengths of 14.16 ± 0.49 cm, 19.03 ± 1.41 cm, 21.33 ± 1.51 cm, and 17.50 ± 0.02 cm, respectively, over the uninoculated control (Figure 5B). The strain ASH19 reported a maximum root length of 41.00 ± 1.17 cm with an increase up to 120% the over uninoculated control. The strains ASH6, ASH20, and ASH22 also showed a better increase in root length of maize up to 38.02 ± 0.94 cm, 38.16 ± 1.62 cm, and 37.33 ± 1.50 cm, respectively, and were non-significant to each other and with strain ASH19 but significantly different from other tested strains and uninoculated control.

The data for maize fresh and dry biomasses are presented in Figures 5, 6 which show a significant increase due to seed inoculation with MSB strains. The maximum shoot fresh biomass of 2.85 ± 0.11 g was reported by strain ASH20 with an increase of up to 275% compared to the uninoculated control and was statistically similar to strain ASH11 which had 2.55 ± 0.11 g of shoot length with an increase of up to 236% compared to the uninoculated control. Strain ASH11 also showed a maximum increase of up to 193% compared to the uninoculated control, as shown by a shoot dry weight of 0.44 ± 0.03 g, which was non-significant to strains ASH6 and ASH20; however, these strains were highly significant to the other tested strains and the uninoculated control. Strains ASH6 and ASH20 also showed better shoot dry biomass of 0.37 ± 0.03 g and 0.38 ± 0.01 g with increases of up to 147 and 153%, respectively, than the uninoculated control. The highest root fresh biomass and root dry biomass were shown by strains ASH19 and ASH22 compared to the uninoculated control. Strain ASH19 reported 2.00 ± 0.03 g of root fresh biomass and 0.58 ± 0.05 g of root dry biomass with an increase up to 292 and 164%, respectively, over uninoculated control. The strains ASH22 reported 1.81 ± 0.02 g of root fresh biomass and 0.65 ± 0.08 g of root dry biomass with increases of up to 255 and 196%, respectively, compared to the uninoculated control. Both of these strains in root fresh and root dry biomass were non-significant, however, they were highly significant to the uninoculated control and other test strains. The strains ASH19, ASH20, and ASH22 reported significantly maximum total fresh biomass of 4.26 ± 0.11 g, 4.43 ± 0.22 g, and 4.25 ± 0.23 g with an increase up to 232, 246, and 232%, respectively, compared to uninoculated control and these strains were statistically similar to each other. The maximum total dry biomass of 0.84 ± 0.04 g, 0.90 ± 0.08 g, 0.90 ± 0.06 g, and 0.99 ± 0.11 g with an increase up to 127, 143, 143, and 168%, respectively, was reported by strains ASH6, ASH11, ASH19, and ASH20, respectively, compared to the uninoculated control (Figures 6A,B).

**FIGURE 7:**
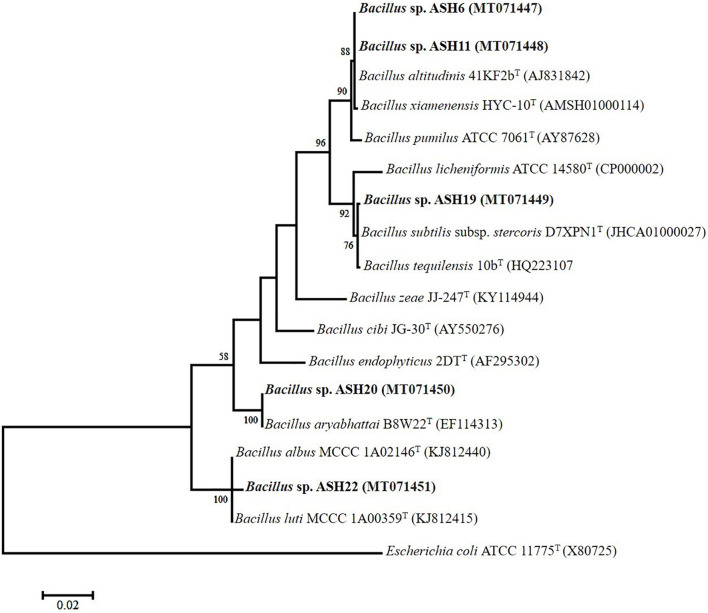
The neighbor-joining phylogenetic tree was produced using multiple alignments of the 16S rRNA gene sequence of *Bacillus* sp. ASH6, *Bacillus* sp. ASH11, *Bacillus* sp. ASH19, *Bacillus* sp. ASH20, and *Bacillus* sp. ASH22 with those of other bacterial strains found in the GenBank database; The 16S rRNA sequences of the identified manganese solubilizing bacterial strains were submitted in the GenBank database and the obtained accession numbers were MT071447, MT071448, MT071449, MT071450, and MT071451, respectively.

The MSB strains significantly promoted Mn concentrations in the shoots compared to the uninoculated control (Figure 6D). The maximum increases of up to 1209% in Mn concentration of 11.26 ± 0.21 μg mL^–1^ in the shoot were reported due to inoculation with strain ASH7 compared to the uninoculated control. Strain ASH8 and ASH20 also reported better Mn concentrations in shoots, with increases of up to 527 and 572%, respectively, than the uninoculated control. The uninoculated control and inoculation of strains with ASH9, ASH10, ASH22, and ASH24 reported the lowest Mn concentrations in the shoots and were non-significant to each other. The Mn concentration in roots was better than the Mn concentration in shoots. Strain ASH20 reported a significantly maximum Mn concentration of 54.91 ± 0.16 μg mL^–1^ in roots with increase of up to 2632% compared to uninoculated control. Strain ASH11 also reported a better increase of up to 2260% in Mn concentration of 47.43 ± 0.31 μg mL^–1^ in the root, than the uninoculated control. Strain ASH17 was not able to promote Mn concentrations in roots and showed statistically similar Mn concentrations in roots to the uninoculated control (Figure 6C). Five best PGP MSB strains *viz*. ASH6, ASH11, ASH19, ASH20, and ASH22 were selected based on their performance to promote maize growth and biomass. A phylogenetic tree of these strains is demonstrated in Figure 7 which shows that all the identified strains belonged to *Bacillus* spp. with accession numbers MT071447, MT071448, MT071449, MT071450, and MT071451, respectively.

### Promotion of Maize Growth and Nutrient Concentrations in Soil Culture Pot Experiment

The MSB strains significantly promoted maize growth and nutrient uptake in soil culture pot experiment with native soil Mn concentrations. The increase in plant growth due to the application of MSB strains was observed in terms of chlorophyll contents, plant height, root length, and shoot and root dry weight of maize (Table 6). The uninoculated control showed minimum chlorophyll contents, plant height, root length, and shoot and root dry weight. The maximum increase of up to 26% in chlorophyll contents was reported by strain ASH19 over the uninoculated control and was statistically similar to strains ASH6 and ASH20 which reported 22 and 23% higher chlorophyll contents as compared to uninoculated control. Strain ASH19 reported a maximum increase up to 18% in plant height compared to the uninoculated control and was statistically similar to other MSB strains except for strain ASH22. Strain ASH19 also reported a significant maximum increase of up to 34% in root length over the uninoculated control. The maximum shoot dry weight was reported by strains ASH11, ASH19, and ASH6, with increases of up to 17, 14, and 12%, respectively, over the uninoculated control. These strains were statistically similar to each other, however, produced significantly different shoot dry weight as compared to uninoculated control. The increase in root dry weight due to the application of MSB strains was statistically similar to each other, however, they showed a statistically higher root dry weight than the uninoculated control. The maximum increase of up to 21% in root dry weight was reported by strain ASH20 over the uninoculated control.

**TABLE 6:** Growth attributes of maize grown with Mn solubilizing bacterial strains under soil cultured pot trial.

Mn-solubilizing Strains	Chlorophyll Contents (SPAD value)	Plant height (cm)	Root length (cm)	Shoot dry weight (g)	Root dry weight (g)
Control	42.25 ± 0.35^d^	144.00 ± 2.64^c^	68.50 ± 0.57^e^	34.30 ± 0.30^c^	8.85 ± 0.35^b^
ASH6	51.53 ± 0.51^abc^	170.00 ± 2.41^ab^	77.00 ± 1.41^d^	38.50 ± 0.50^ab^	10.05 ± 0.45^a^
ASH11	49.56 ± 0.76^c^	169.00 ± 1.87^ab^	87.50 ± 1.58^b^	40.00 ± 1.11^a^	10.40 ± 0.79^a^
ASH19	53.37 ± 0.47^a^	170.50 ± 2.45^a^	92.00 ± 1.44^a^	39.00 ± 1.01^a^	10.40 ± 0.45^a^
ASH20	52.12 ± 0.42^ab^	170.00 ± 2.16^ab^	86.00 ± 1.17^b^	35.50 ± 0.56^c^	10.70 ± 0.72^a^
ASH22	50.50 ± 0.50^bc^	167.50 ± 1.98^b^	80.50 ± 0.77^c^	36.50 ± 0.67^bc^	10.20 ± 0.41^a^
LSD	2.00	3.20	3.39	2.38	0.70

*Maize seeds inoculated with MSB were grown in soil cultured pot trial along with native soil Mn. The chlorophyll contents in terms of SPAD values were observed from the second top leaf of maize at the cob formation stage, however, the other growth attributes were recorded at the time of physiological maturity; Data presented here are the mean of three replications ± standard error; the means in a vertical line for each attributes sharing the common letters were considered statistical similar to each other and the least significant difference (LSD) test was performed at 5% (*p* ≤ 0.05) probability level.*

**FIGURE 8:**
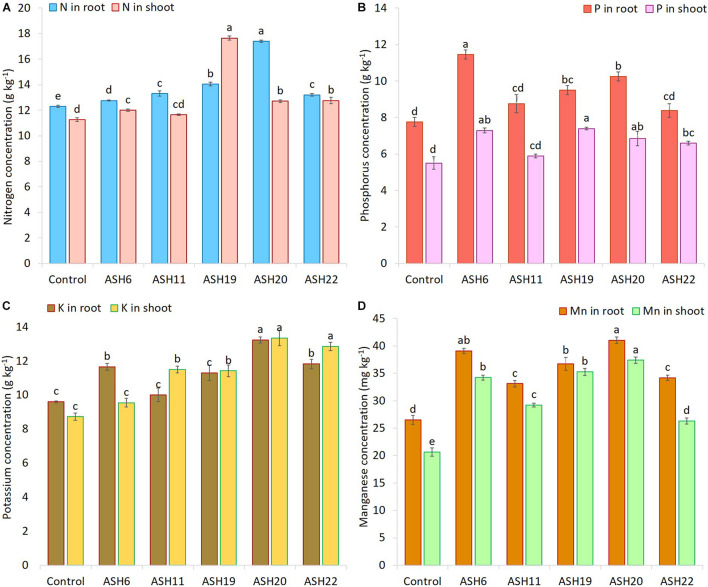
The concentration of nitrogen **(A)**, phosphorus **(B)**, potassium **(C)**, and manganese **(D)** in maize roots and shoots grown with Mn solubilizing bacterial (MSB) strains; maize seeds inoculated with MSB were grown up to physiological maturity in earthen pots filled with sandy loam textural soil having native 14.36 mg kg^–1^ extractable manganese and 26.90 mg kg^–1^ total manganese; the uninoculated control had no inoculum and contains only broth; data presented are the mean of three replications ± standard error; the means sharing same alphabetical letters were considered non-significant to each other and least significant difference (LSD) test was performed at 5% (*p* ≤ 0.05) probability level.

**FIGURE 9:**
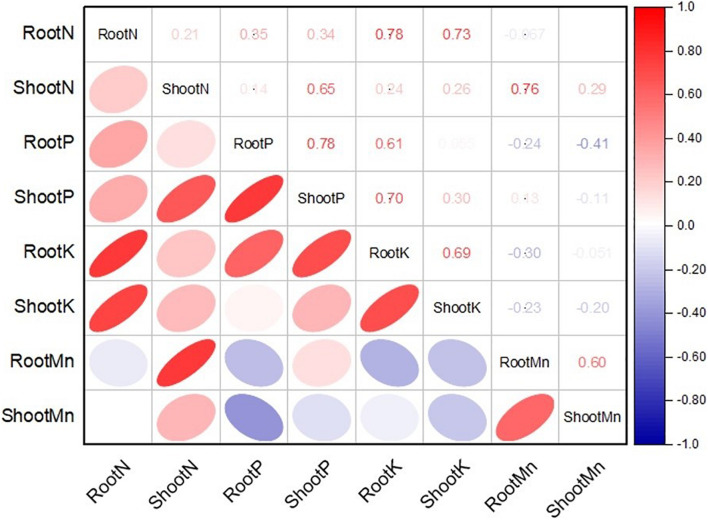
Pearson correlations analysis among nitrogen, phosphorus, potassium, and manganese concentration in roots and shoots of maize plant treated with Mn solubilizing bacterial (MSB) strains under soil culture pot trial; RootN, root nitrogen; ShootN, shoot nitrogen; RootP, root phosphorus; ShootP, shoot phosphorus; RootK, root potassium; ShootK, shoot potassium; RootMn, root manganese; ShootMn, shoot manganese.

The application of MSB strains improved nutrient uptake in terms of N, P, and K concentrations in maize roots and shoots as compared to the uninoculated control (Figure 8). The uninoculated control reported a minimum concentration of these nutrients in maize roots and shoots. Strain ASH20 reported a maximum increase of up to 42% in N concentration in maize roots compared to the uninoculated control, while, a maximum increase of up to 57% in N concentration in maize shoots was reported by strain ASH19 over the uninoculated control (Figure 8A). The maximum increase of up to 48% in P concentration in maize roots over the uninoculated control was reported by strain ASH6 while strain ASH20 followed by ASH6 reported maximum increases of up to 35 and 33%, respectively in P concentration in the shoot compared to the uninoculated control (Figure 8B). A higher increase in K concentration in maize roots of up to 38% was observed through inoculation with strain ASH20 compared to the uninoculated control. The maximum increase up to 53% in K concentration in maize shoots was reported by strain ASH20 followed by strain ASH22, which reported 47% more K concentration in the shoots compared to the uninoculated control (Figure 8C). The application of MSB strains significantly promoted Mn concentrations in maize roots and shoots compared with the uninoculated control (Figure 8D). The maximum increase in Mn concentrations in maize roots up to 55% was reported by strain ASH20 followed by strain ASH6, which showed a 48% increase in Mn concentration in roots compared to the uninoculated control. Strain ASH20 also reported a maximum increase of up to 81% in Mn concentration in maize shoots followed by strain ASH19, which reported a 71% higher Mn concentration than the uninoculated control. Correlation analysis revealed a positive association among nutrients contents in soil culture pot trial (Figure 9). Meanwhile, a negative correlation of Mn concentration in roots and shoots was observed with P concentration in roots and K concentration in shoots. The Mn concentration in roots was also negatively correlated to K concentration in roots.

## Discussion

Manganese deficiency can be a serious limiting factor for plant growth in calcareous sandy soils with high pH (Husted et al., 2005). Plants tend to obtain Mn by establishing a symbiosis with Mn-reducing soil bacteria that solubilize MnO_2_. In the present study, MSB strains were isolated from the maize rhizosphere and identified as *Bacillus* spp. through 16S rRNA gene sequencing. These strains caused a significant increase in plant growth and Mn concentration in maize seedlings through the solubilizing of an insoluble source of Mn. To the best of our knowledge, this is the first study regarding the characterization and application of MSB strains for plant growth promotion and Mn uptake.

In the present study, bacterial strains isolated from maize rhizosphere showed the ability to solubilize Mn on nutrient agar media amended with 50 mM MnO_2_. Similar to our work, Kasana et al. (2008) also flooded Gram’s iodine solution on carboxymethylcellulose agar media for the determination of cellulase production by bacteria and reported very dominant clearing zones around the colonies. In the present study, thirteen strains showed a predominant solubilization zone around the bacterial growth, and the strains *Bacillus* sp. ASH4, *Bacillus* sp. ASH10, and *Bacillus* sp. ASH20 showed the highest value for MSI and MSE (Table 1). Mn solubilization by numerous microorganisms has also been reported in various studies (Madgwick, 1991; Wei et al., 2012; Sanket et al., 2017). Madgwick (1991) reported reductive solubilization of MnO_2_ tailings by four microbial isolates *viz*. *Achromobacter* sp., *Aspergillus niger*, *Enterobacter cloacae*, and *Enterobacter agglomerans*. Similarly, Sanket et al. (2017) reported the ability of four acidophilic bacterial strains *viz*. *Enterobacter* sp. AMSB1, *B. cereus* AMSB3, *Bacillus nealsonii* AMSB4, and *Staphylococcus hominis* AMSB5 to solubilize Mn on MnO_2_-amended nutrient agar media.

In the present study, *Bacillus* strains solubilized Mn concentrations ranging from 3.56 ± 0.13 μg mL^–1^ to 10.73 ± 0.59 μg mL^–1^ (Table 1). *Bacillus* sp. ASH6 and *Bacillus* sp. ASH20 demonstrated the maximum solubilized concentration of Mn, which may be due to its ability to reduce Mn(IV) and Mn(III) into Mn(II). Enzymatic reduction of Mn(IV) oxidized Mn(II) serves as a terminal electron acceptor for aerobic and facultative anaerobic bacteria in the form of respiration and reduced Mn(II) concentration to satisfy their nutritional needs (Bromfield and David, 1976). The extracellular heme or flavin enzyme called cellobiose dehydrogenase plays a role in the redox cycling of Mn in nature and can reduce insoluble MnO_2_ into soluble Mn(II) (Roy et al., 1994). Organic acids, including formic, oxalic, pyruvic, salicylic, citric, and malic acids cause a significant reduction in soil pH, which plays an important role in the conversion of MnO_2_ into the plant-available Mn^2+^ form (Di-Ruggiero and Gounot, 1990; Rusin and Ehrlich, 1995). The production of inorganic compounds, including ferrous iron and sulfide, during anaerobic respiration or the production of H_2_O_2_ during aerobic respiration also causes a reduction in Mn(IV) (Rusin and Ehrlich, 1995). The tested MSB strains were also positive for qualitative and quantitative solubilization of P and Zn (Tables 2, 3). However, only six out of thirteen MSB strains were positive for K solubilization (Table 4). The solubilization of such insoluble minerals by MSB strains might be due to the production of organic acids, including 2-ketogluconic, acetic, aspartic, citric, fumaric, gluconic, glutamic, glycolic, glyoxylic, isobutyric, isovaleric, itaconic, lactic, maleic, malic, malonic, oxalic, propionic, succinic, tartaric, and α-ketobutyric acids (Vyas and Gulati, 2009; Etesami et al., 2017; Nath et al., 2017; Wei et al., 2017; Dinesh et al., 2018; Mumtaz et al., 2019). Such organic acid production by various types of bacteria is autonomous of their genetic relatedness, and each strain has its specific capability of producing organic acids during the solubilization of inorganic minerals (Vyas and Gulati, 2009).

In the current study, eight out of thirteen MSB strains were able to produce IAA in the presence of L-tryptophan (Table 5). An increase in IAA production may promote primary root elongation and the formation of lateral and adventitious roots (Xie et al., 1996). Recently, Panigrahi et al. (2020) reported variation in the production of IAA by endophytic *E. cloacae* to strain MG00145 isolated from *Ocimum sanctum* and significantly promoted the growth of various crops, including *Oryza sativa*, *Arachis hypogaea*, *Vigna mungo*, and *Brassica campestris* var. toria. In the current study, all the MSB strains showed their ability to produce NH_3_, while, eight out of thirteen MSB strains showed siderophore production, which may promote plant growth by providing N and Fe to plants and could be involved in biocontrol by limiting Fe availability to pathogenic fungi (Sayyed et al., 2013). The application of rhizobacteria gradually increases microbial enzymatic activities and shows a substantial increase in plant growth. In the present study, the majority of MSB strains tested positive for protease, amylase, lipase, and catalase activities. These are hydrolytic enzymes that could play important roles in improving soil fertility by enhancing the decomposition of various organic residues of plant and animal origins.

The results of pot trials under axenic sand culture and soil culture conditions revealed that the tested MSB strains showed their ability to promote maize growth in terms of shoot and root length, and shoot and root fresh and dry biomass in the presence of insoluble Mn; however, the strains ASH7, ASH10, and ASH12 were not able to promote maize growth under sand culture conditions and showed statistically lower maize growth than the uninoculated control (Figure 5). Significant variation among the MSB strains was observed, while, *Bacillus* sp. ASH6, *Bacillus* sp. ASH11, *Bacillus* sp. ASH19, *Bacillus* sp. ASH20, and *Bacillus* sp. ASH22 reported a maximum increase in the growth of maize seedlings compared to other tested strains. The findings of the current study are in line with our previous study about the application of Zn-solubilizing *Bacillus* strains to promote maize growth (Mumtaz et al., 2017). Similarly, Naseer et al. (2020) reported an increase in rice germination, growth, and vigor index in pot trials due to inoculation with Zn solubilizing *Bacillus* strains *viz*. *Bacillus megaterium* AN24, *Bacillus aryabhattai* AN30, *B. megaterium* AN31, and *B. megaterium* AN35. Ahmad et al. (2020) inoculated cotton seeds with seven PSB and seven ZSB strains in pot trials and reported a significant increase in growth attributes over the control. They reported the best outcome in terms of co-inoculation of *Bacillus subtilis* IA6 + *Bacillus* sp. IA16 followed by *Paenibacillus polymyxa* IA7 + *B. aryabhattai* IA20. Similarly, inoculation with P solubilizing endophytic *P. polymyxa* ANM59 and *Paenibacillus* sp. ANM76 also showed promising results to improve the growth and nodulation of chickpea (Ahmad et al., 2019).

In the present study, the increase in maize growth due to inoculation with MSB strains in the presence of insoluble Mn compounds in the sand as well as soil culture pot experiments might be due to their ability to solubilize Mn and other minerals including P, K, and Zn, and their ability to produce siderophores, IAA, NH_3_, and perform various enzymatic activities which we determined in the current *in vitro* and *in vivo* studies. Our findings are also supported by previous studies reported by Mumtaz et al. (2017, 2019), Ahmad et al. (2019, 2020), and Panigrahi et al. (2020). The application of such beneficial mineral-solubilizing bacteria could improve nutrient availability in root zones and enhance their uptake in plants (Kumawat et al., 2017; Mumtaz et al., 2018, 2020). The production of IAA by MSB strains could also have a possible role in increasing maize growth in pot trials. Bacterial IAA induces plant growth and development by affecting cell division, initiation, and elongation of lateral and adventitious roots (Tsavkelova et al., 2006; Spaepen et al., 2007). Bacterial IAA also increases root surface area and root length, as we observed in the current study, as demonstrated in Figure 4 which helps plants absorb more nutrients from the soil (Patten and Glick, 2002). The current study also revealed the higher uptake of N, P, K, and Mn in the shoots and roots of maize due to inoculation with MSB strains compared to the uninoculated control (Figures 6, 8). The increase in N uptake in maize roots and shoots might be due to the ability of MSB strains to fix atmospheric N and increase its uptake into maize root and shoot tissues. The current study demonstrated the ability of MSB strains to solubilize P (Table 2) and K (Table 4) which also helped the mobilization of P and K from soil solution to maize root and shoot tissues (Figure 8). Mn uptake in roots and shoots demonstrated the strong power of MSB to solubilize and mobilize the stability of MnO_2_ under axenic sand culture as well as soil culture conditions and increase its translocation from root to shoot compared to the uninoculated control. Similarly, the literature has reported that Mn-reducing bacteria, including *Arthrobacter* and *Variovorax* spp. promoted plant growth and Mn uptake in wheat and soybean (Marschner et al., 1991; Nogueira et al., 2007).

## Conclusion

The present study concluded that *Bacillus* spp. strains ASH6, ASH11, ASH19, ASH20, and ASH22 showed strong power to solubilize Mn and improve maize growth attributes and Mn uptake in maize roots and shoots. These strains possess multiple PGP attributes and could be attractive inoculants to improve maize productivity by addressing nutrient deficiencies. Such prospective bioinoculants could address the issue of Mn as well as P, K, Zn, and Fe deficiencies in plants in calcareous soil after further evaluation. The current findings suggest that researchers evaluate MSB strains to study their genetic and molecular mechanisms of Mn solubilization. Further evaluation of the promising MSB strain’s ability to increase maize and other crop productivity should be conducted under laboratory, pot, and field conditions.

## Data Availability Statement

The datasets presented in this study can be found in online repositories. The names of the repository/repositories and accession number(s) can be found in the article/Supplementary Material.

## Author Contributions

MM and AI conceived and designed the experimental strategies. AI and HM performed all experiments and data analysis. MA provided the study materials, reagents, research resources, and facilitated critical review on pre-publication stages. MM created and presented the published work specifically writing the initial and final draft and performed the revision. AZ helped in the identification of bacterial strains and performed the phylogenetic analysis. MA and MS oversight the research activity, planning, and execution. XW and WW provided financial support for the publication of this article. All authors contributed to the article and approved the submitted version.

## Conflict of Interest

The authors declare that the research was conducted in the absence of any commercial or financial relationships that could be construed as a potential conflict of interest.

## Publisher’s Note

All claims expressed in this article are solely those of the authors and do not necessarily represent those of their affiliated organizations, or those of the publisher, the editors and the reviewers. Any product that may be evaluated in this article, or claim that may be made by its manufacturer, is not guaranteed or endorsed by the publisher.
