# Supplementary material for: Insights Into Manganese Solubilizing Bacillus spp. for Improving Plant Growth and Manganese Uptake in Maize
Source: Front Plant Sci. 2021 Nov 2;12:719504. doi: 10.3389/fpls.2021.719504 (PMC8593242; doi:10.3389/fpls.2021.719504)
Supplement: Supplementary file 1 [file Data_Sheet_1.docx]

Supplementary Material

**Supplementary Table 1: Colony morphology of manganese solubilizing bacterial strains isolated from maize**

| **Strains** | **Color** | **Form** | **Elevation** | **Margin** | **Appearance** |
| --- | --- | --- | --- | --- | --- |
| ASH1 | Dark yellow | Irregular | Raised | Entire | Smooth shiny |
| ASH2 | Yellow | Circular | Raised | Entire | Smooth |
| ASH3 | Light pink | Circular | Flat | Entire | Smooth shiny |
| ASH4 | White | Circular | Raised | Entire | Smooth |
| ASH5 | Yellow | Circular | Raised | Entire | Smooth |
| ASH6 | Light yellow | Circular | Raised | Entire | Smooth |
| ASH7 | Creamy white | Circular | Raised | Entire | Smooth |
| ASH8 | Off-white | Circular | Flat | Entire | Smooth |
| ASH9 | White | Irregular | Flat | Entire | Rough |
| ASH10 | Light-yellow | Circular | Raised | Entire | Smooth |
| ASH11 | Yellow | Circular | Raised | Entire | Smooth |
| ASH12 | White | Irregular | Raised | Undulate | Rough |
| ASH14 | White | Filamentous | Umbonate | Filamentous | Rough |
| ASH15 | Off-white | Circular | Raised | Entire | Smooth shiny |
| ASH16 | Creamy white | Punctiform | Raised | Entire | Smooth |
| ASH17 | White | Irregular | Umbonate | Undulate | Rough |
| ASH18 | Light yellow | Irregular | Flat | Entire | Smooth |
| ASH19 | White | Irregular | Raised | Entire | Rough |
| ASH20 | Yellow | Circular | Raised | Entire | Smooth |
| ASH22 | Creamy white | Irregular | Raised | Entire | Smooth |
| ASH24 | Light yellow | Circular | Raised | Entire | Smooth |


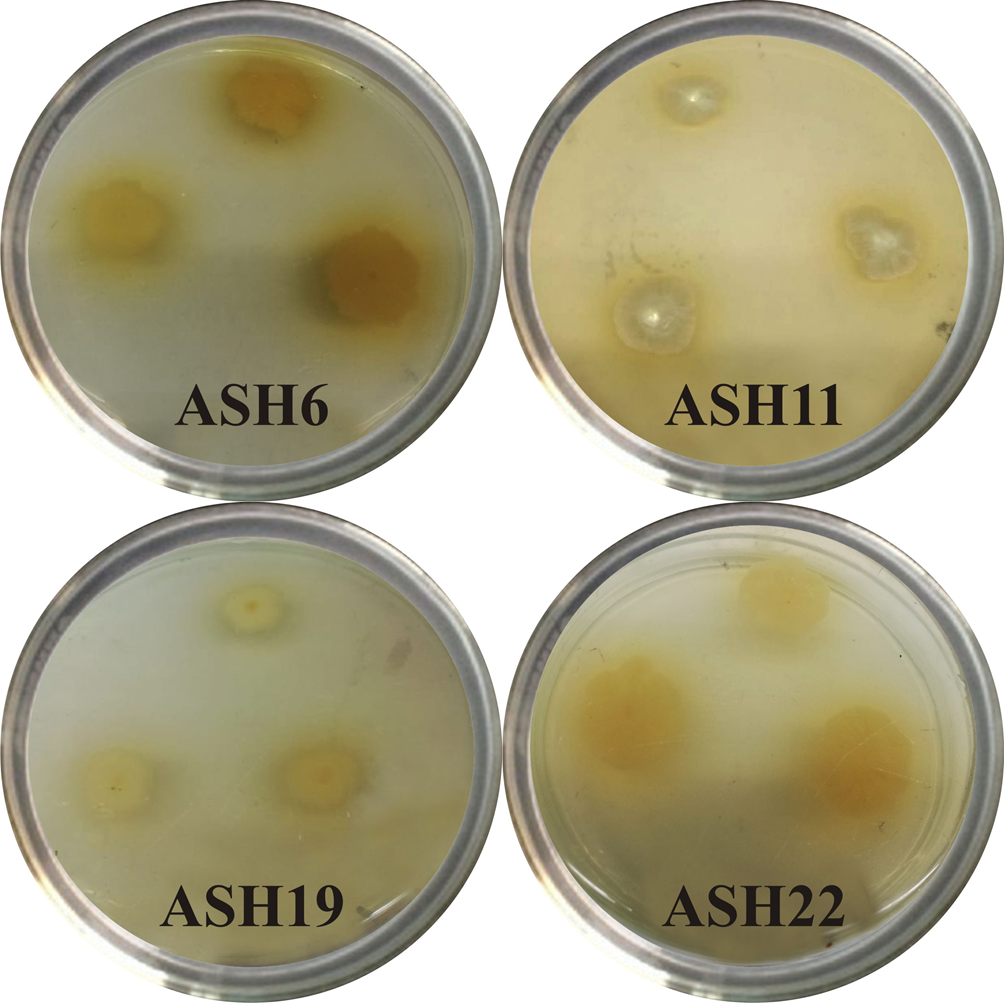


**Supplementary Figure 1.** Solubilization of manganese oxide (MnO2) by manganese solubilizing bacterial strains ASH6, ASH11, ASH19, and ASH22 on MnO2-amended nutrient agar without iodine staining.


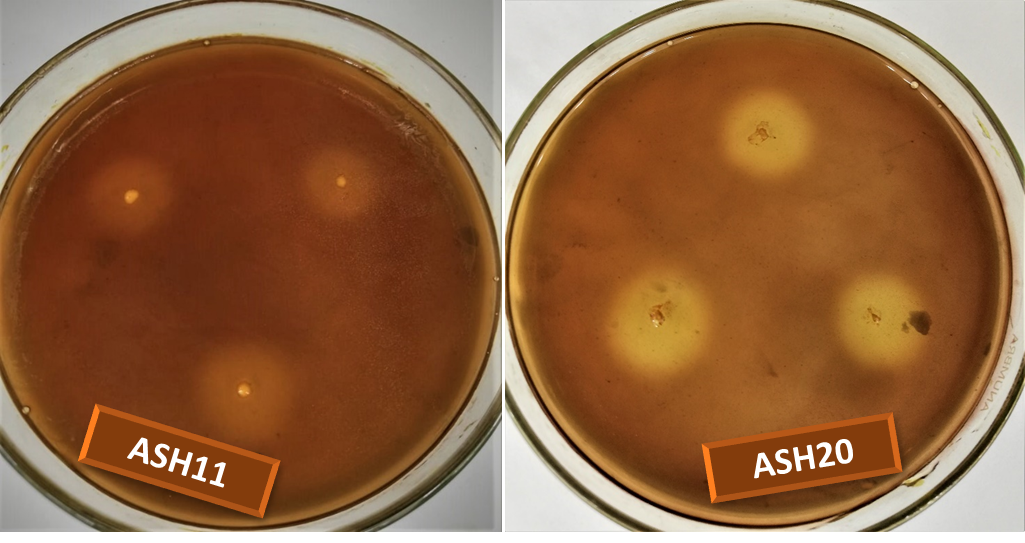


**Supplementary Figure 2.** Siderophores production by manganese solubilizing bacterial strains.


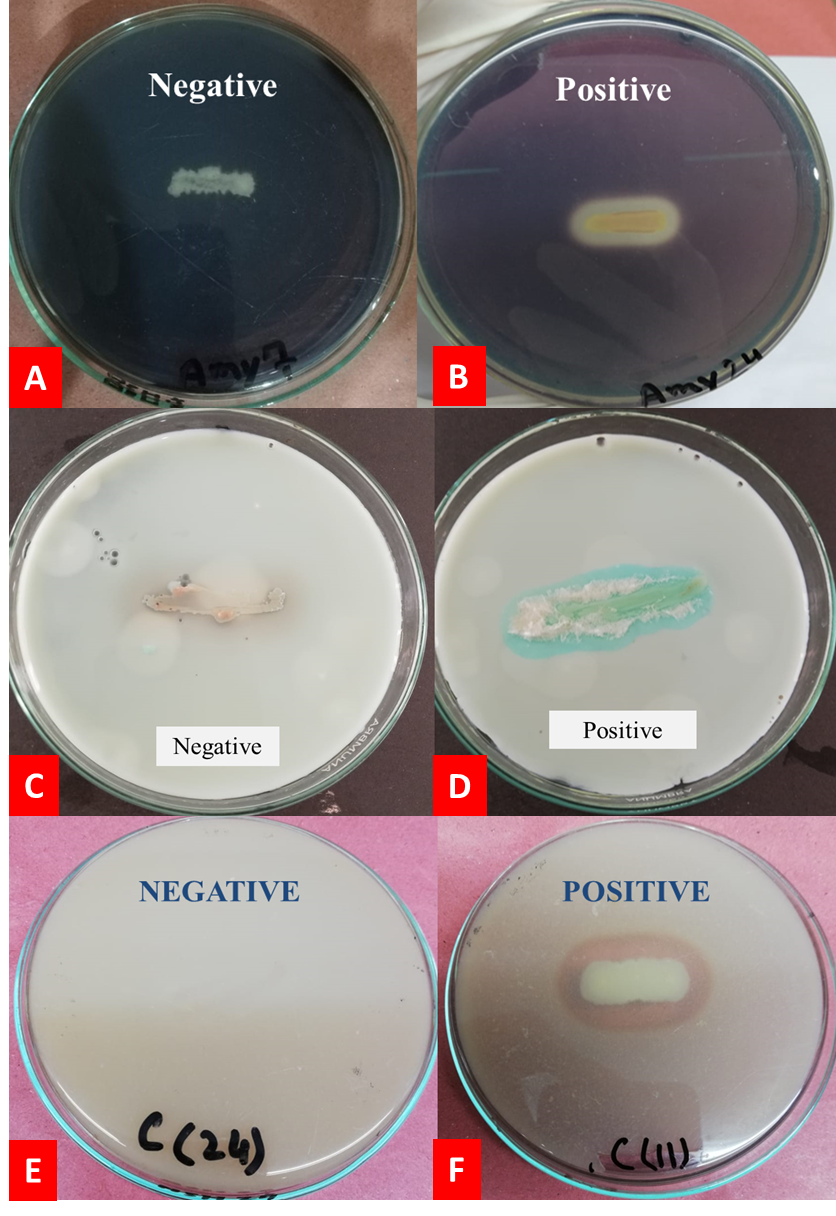


**Supplementary Figure 3.** The amylase, lipase and protease activities were performed by manganese solubilizing bacterial strains; the amylase activity trait was considered negative (A) due to absence of halo zone and positive (B) when halo zone was present around bacterial growth; the lipase activity was absent (C) when no halo zone was observed around the bacterial growth while lipase positive (D) strains showed halo zone around colony growth; proteases negative (E) strains was unable to degrade the casein substrate and showed absence of halo zone while proteases positive (F) strains showed their ability to degrade casein substrate.


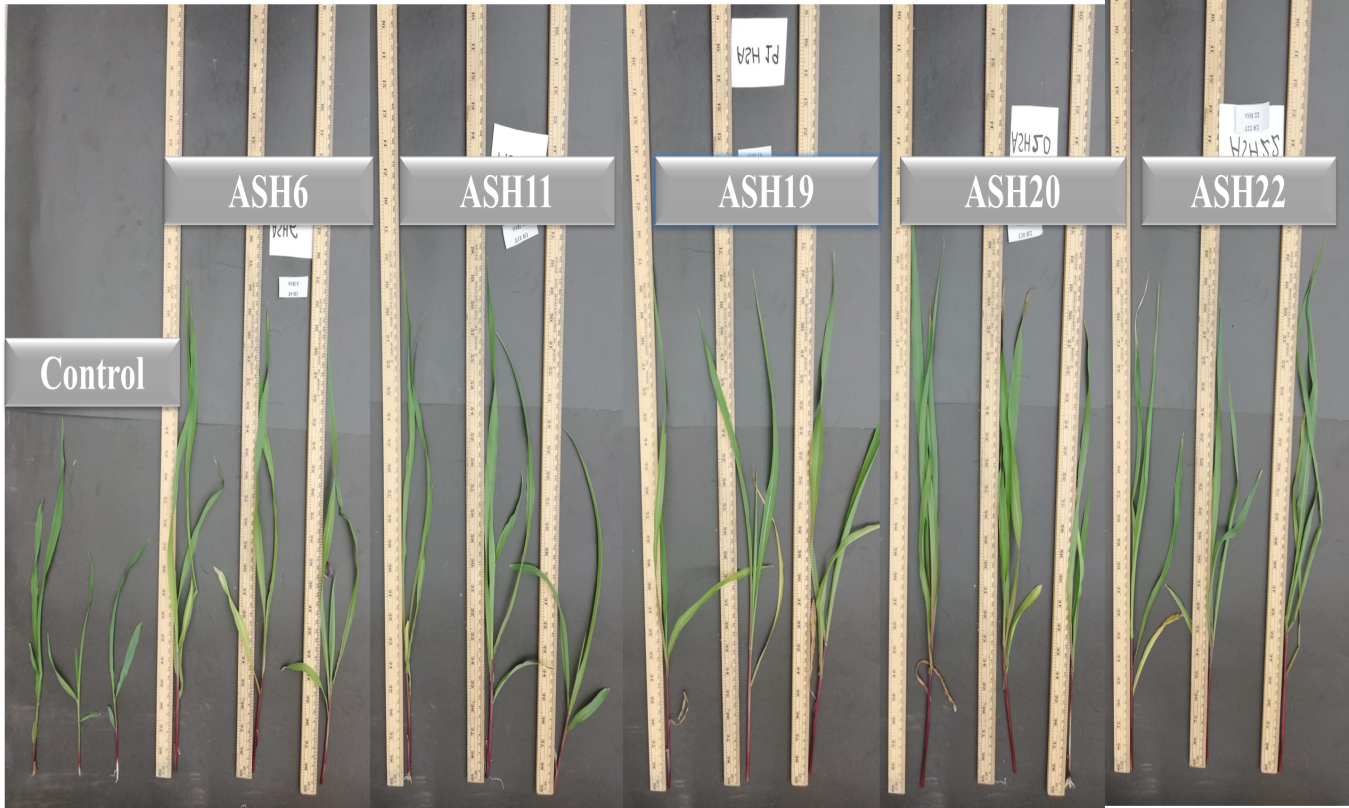


**Supplementary Figure 4.** Effect of manganese solubilizing bacterial strains on shoot length of maize under pure sand culture amended with manganese oxide in pot experimental conditions.
